# Eye contact avoidance in crowds: A large wearable eye-tracking study

**DOI:** 10.3758/s13414-022-02541-z

**Published:** 2022-08-22

**Authors:** Roy S. Hessels, Jeroen S. Benjamins, Diederick C. Niehorster, Andrea J. van Doorn, Jan J. Koenderink, Gijs A. Holleman, Yentl J. R. de Kloe, Niilo V. Valtakari, Sebas van Hal, Ignace T. C. Hooge

**Affiliations:** 1 Experimental Psychology, Helmholtz Institute, Utrecht University, 3584CS Utrecht, The Netherlands; 2 Social, Health and Organisational Psychology, Utrecht University, Utrecht, The Netherlands; 3 Lund University Humanities Lab, Lund University, Lund, Sweden; 4 Department of Psychology, Lund University, Lund, Sweden

**Keywords:** Eye contact, Crowd navigation, Eye tracking, Wearable, Gaze

## Abstract

Eye contact is essential for human interactions. We investigated whether humans are able to avoid eye contact while navigating crowds. At a science festival, we fitted 62 participants with a wearable eye tracker and instructed them to walk a route. Half of the participants were further instructed to avoid eye contact. We report that humans can flexibly allocate their gaze while navigating crowds and avoid eye contact primarily by orienting their head and eyes towards the floor. We discuss implications for crowd navigation and gaze behavior. In addition, we address a number of issues encountered in such field studies with regard to data quality, control of the environment, and participant adherence to instructions. We stress that methodological innovation and scientific progress are strongly interrelated.

## Introduction

Eye contact is considered to serve essential functions in face-to-face interactions (Argyle & Dean, 1965). Maintaining eye contact is thought to be a key to teacher competence in teacher-student interactions (Smidekova, Janik, Minarikova, & Holmqvist, 2020), and eye contact is further relevant to the study of social anxiety and/or autism spectrum disorders (Hessels, Holleman, Cornelissen, Hooge, & Kemner, 2018a; Senju & Johnson, 2009; Wieser, Pauli, Alpers, & Mühlberger, 2009), patient-clinician interactions (Jongerius et al., 2021; MacDonald, 2015) and social robotics (Kiilavuori, Sariola, Peltola, & Hietanen, 2021). Although eye contact may be defined and operationalized in various ways (Jongerius, Hessels, Romijn, Smets, & Hillen, 2020)—it may e.g. be considered from a phenomenological standpoint (Heron, 1970; Honma, Tanaka, Osada, & Kuriyama, 2012)—a common operationalization of eye contact is mutual looking at the eyes and/or face by two interactors (e.g. Kleinke 1986), often estimated using eye tracking technology (Jongerius et al., 2020).

While the processing of eye contact (e.g. operationalized by having people look at schematic faces or photographs of faces looking straight ahead) has long been considered a model system for human social interaction and communication (Senju & Johnson, 2009), several researchers have raised the concern that such simplified operationalizations may misrepresent how eye contact and other phenomena of social attention support behavior in social interactions (e.g. Kingstone, 2009; Risko, Laidlaw, Freeth, Foulsham, & Kingstone, 2012; Risko, Richardson, & Kingstone, 2016). Recent advances in eye-tracking technology, and particularly the widespread availability of wearable eye-tracking glasses, are seen as providing the methodological tools to address this problem (Pérez-Edgar, MacNeill, & Fu, 2020; Shamay-Tsoory & Mendelsohn, 2019; Valtakari et al.,, 2021). Wearable eye trackers bring the promise of being able to estimate when and how long humans look at the face or body of others or make eye contact^1^ while they engage in various activities of daily life. In our experience however, the practical feasibility of this kind of research and the ease of interpretation of the results in light of psychological theories are often overestimated (Hessels, Niehorster, Holleman, Benjamins, & Hooge, 2020b; Holleman, Hooge, Kemner, & Hessels, 2020b, 2020c; Niehorster et al., 2020b), particularly by researchers new to eye tracking. Unfortunately, little practical advice on conducting wearable eye-tracking studies in various ‘real world contexts’ exists.

In this study, we determine empirically whether humans can avoid eye contact while navigating through crowds or whether looking at other people’s faces or eyes is automatic and difficult to override. In addition, we outline several practical problems encountered when conducting a wearable eye-tracking study on eye contact in a live festival setting. We address the background for each in turn.

### Eye contact in crowds

Eye contact in face-to-face interactions has been a thriving area of study in the 1960s-1980s. Eye contact was studied for its regulatory functions in face-to-face interactions, for example with regard to turn-taking, or maintaining an appropriate level of intimacy or emotionality (e.g. Argyle and Dean, 1965; Kendon, 1967; Kleinke, 1986). Given that abnormal or inadequate eye contact may be diagnostic for Autism Spectrum Disorder or Social Anxiety Disorder (American Psychiatric Association, 2013), it seems relevant to understand what functions eye contact may serve across various social contexts. Here we are concerned with what role eye contact plays in human crowds. Human crowds are interesting because they may contain many encounters of a fleeting nature, some of which may lead to some form of further interaction (e.g. a conversation or brief exchange of smiles) whereas many do not.

Since the pioneering work of Buswell (1935) and Yarbus (1967), it has frequently been reported that humans have a bias for looking at other people, their faces and their eyes (see Hessels, 2020, for a recent review). Based on this, one might expect that humans tend to make eye contact with others as they navigate their social environments. However, it appears that whether the faces and eyes of others are fixated depends critically on whether there is potential for interaction. For example, Laidlaw, Foulsham, Kuhn and Kingstone (2011) showed that a live confederate in a waiting room was hardly looked at while a videotaped confederate was, which the authors suggested was due to the live confederate potentially engaging the participant in social interaction (cf. the phenomenon of ‘civil inattention’ described by Goffman, 1966). Thus, the question beckons to what degree human crowds actually afford interaction.

Intuitively, one would expect that there is substantial potential for interaction in crowds. While walking through a busy street in one’s home town, one may readily encounter friends or neighbors that may engage one in interaction. Obviously, it depends on the nature of the crowd and the context to what degree interaction is possible. A busy street in one’s home town may differ substantially in that respect from a crowd during a morning commute at a large train station or crowds during riots. Empirical eye-tracking research on the topic is scarce, however, which Berton, Hoyet, Olivier and Pettré (2018) point out may be because studies with “users wearing eye-trackers in real environments ... can be difficult to organize in real crowds because of technical, human, and experimental organization” (p. 1). In contrast, several studies have been conducted on gaze behavior during passing encounters. Foulsham, Walker and Kingstone (2011), for example, showed that humans were likely to gaze more at a passerby on a university campus when they were far away than at a close interpersonal distance. They suggested that humans may avoid a potential interaction with the passerby by looking away at the closer distance. In a recent study, Hessels et al., (2020a) showed that when and where humans look on the body of a passerby depends both on the interpersonal distance and the behavior carried out by the passerby. Participants’ gaze seemed to be directed at the body part of the passerby that was currently relevant, e.g. based on whether they handed out a flyer, looked at their phone or waved at the participant. Interestingly, substantial individual differences were observed for where participants tended to fixate on the passerby (upper body, arms, or lower body) which were consistent across the various passersby. Thus, whether humans look at the faces of others in a brief encounter may depend on the individual, the interpersonal distance and what the other may afford in terms of social interaction.

A crowd obviously consists of many more people than a single passerby, and one can wonder whether looking at others or making eye contact is necessary to navigate crowds successfully. In a study on collision avoidance with a single confederate pedestrian, Croft and Panchuk (2018) reported that looking at the pedestrian predicted passing behind that person, while Jovancevic-Misic and Hayhoe (2009) reported that potential ‘colliders’ were preferentially fixated after only few encounters. Thus, looking at others may be relevant for collision avoidance. This was corroborated in a virtual reality study by Meerhoff, Bruneau, Vu, Olivier and Pettré (2018), who showed that gaze was often directed to other agents that were likely to collide with the controlled virtual agent and which were subsequently avoided. In a study with real crowds of various sizes (6, 12 and 20 walkers), Hessels, van Doorn, Benjamins, Holleman and Hooge (2020c) showed that gaze behavior during crowd navigation is task dependent and not every fixation is strictly necessary for navigating the crowd. When participants were asked to seek out social affordances (i.e. determine whether people in the crowd made eye contact or not), more fixations were directed at the faces of others, which came at the cost of looking at bodies. Thus, looking at the bodies of others may be relevant for navigating crowds successfully, but there is substantial flexibility in where gaze is allocated on the body of others. However, whether humans can avoid looking at the eyes or faces of others while navigating crowds is an open question.

The question of whether humans can navigate crowds without making eye contact is theoretically interesting for several reasons. First, previous research has shown that faces automatically attract and maintain attention (Bindemann, Burton, Hooge, Jenkins, & de Haan, 2005; Langton, Law, Burton, & Schweinberger, 2008) and are preferentially looked at (e.g. Birmingham, Bischof, & Kingstone, 2009; Frank, Vul, & Saxe, 2012; Johnson, Dziurawiec, Ellis, & Morton, 1991). One may wonder whether this assumed automaticity is displayed or can be overridden while navigating crowds. Although it has been shown that looking at others depends on the social context (e.g. Laidlaw et al.,, 2011), Fotios, Uttley and Yang (2015) suggest that “there is a bias towards fixation on other people when they appear in a scene and this may be regardless of their apparent movement or behaviour” (p. 157-158). Notably, they made this suggestion in the context of a wearable eye-tracking study on outdoor pedestrian navigation. Second, gaze behavior during crowd navigation may be seen as a ‘soft constraints’-problem (e.g. Gray, Sims, Fu, & Schoelles, 2006). That is, fixations on other people’s faces may help navigate crowds, but other strategies may be adopted too. Uncovering when and how gaze can be flexibly allocated may help us understand the visual constraints in crowd navigation. Finally, whether humans can navigate crowds without making eye contact may help uncover generic and specific patterns of gaze behavior during fleeting social encounters.

### Wearable eye tracking for the study of social behavior

While the topic is clearly interesting from a scientific standpoint, there are few studies on gaze behavior in real crowds, which Berton et al., (2018) point out may be because of technical, human, or experimental difficulties. Technically, much has changed since the early days of wearable eye tracking. For example, pioneering studies by Land (1993), Land and Lee (1994) and Pelz and Canosa (2001) relied on self-built or self-customized eye trackers or substantial manual calibration and analysis. At present, there are commercially available wearable eye trackers (e.g. Pupil Invisible or the Tobii Pro Glasses 2 and 3) that, for example, allow calibration-free recording or recording using a one-point calibration routine and require minimal setup effort. Thus, it is increasingly easier to record gaze behavior in whatever situations humans find themselves in.

Conducting systematic experiments using crowds may also be difficult from the perspective of human or experimental organization. If one wants full control of crowd size, behavior in crowds, and so forth, one needs a large group of volunteers to conduct the study or one needs to resort to crowds in virtual reality (see e.g. Hessels et al.,, 2020c; Rio & Warren, 2014; Rio, Dachner, & Warren, 2018), which isn’t always feasible. Conducting observations with crowds as one encounters them in unconstrained environments, however, may come at the cost of a complete lack of control. But what can one expect in such situations? Surprisingly little is written about this topic. Yet, a number of the present authors are experienced teachers in eye-tracking courses, and we often meet researchers new to eye tracking with grand expectations of the wearable eye tracker, precisely for these unconstrained environments where there is little control. We know many examples of wearable eye-tracking recordings being conducted in real classrooms, supermarkets or other locations, which end up unanalyzed on a hard drive. In our experience, this is at least partly due to an underestimation of the data-analysis and interpretation problem, and little knowledge about what may go wrong in such unconstrained environments. Thus, we believe that practical insights on conducting wearable eye-tracking studies in unconstrained environments are useful to many researchers working with the technique.

One may wonder why we do not separate our scientific findings from the practical considerations, for example in separate outlets. We believe that the two are intimately intertwined. The practical considerations are necessary in our opinion to delineate the limits of the state-of-the-art and ensure scientific progress in this relatively young research field. Conversely, the practical consideration and issues we ran into are best appreciated in the context of the research question we set out to answer.

### The present study

We conducted a study on eye contact avoidance during crowd navigation at the Betweter festival (roughly translated as Smartass festival), an annual popular science festival in the event hall Tivoli Vredenburg, Utrecht, the Netherlands. While one might consider a festival as an inappropriate location for scientific research, our group has conducted experiments at this festival for several years, which has resulted in multiple scientific publications (e.g Holleman, Hessels, Kemner, & Hooge, 2020a; Hooge, Holleman, Haukes, & Hessels, 2019b).

Participants were fitted with a wearable eye tracker and were asked to walk a round across the festival grounds. Half of the participants were furthermore instructed to avoid eye contact with anyone. We had the following research questions:

1. Can people avoid looking at the faces of others while navigating crowds when instructed to avoid eye contact?
2. Does the instruction to avoid eye contact lead to altered gaze and/or walking behavior?
3. What unforeseen circumstances can one encounter in a wearable eye-tracking study with real crowds?

## Methods

### Participants

People volunteered themselves for the experiment at the Betweter festival by approaching one of the experimenters. A total of 62 volunteers participated in the experiment, with a mean age of 36 years (*sd* = 14 years, range 19–69 years), 40 of whom were female and 22 male. Most participants reported normal or corrected-to-normal vision. However, 8 out of the 62 participants removed their glasses for the experiment as the Tobii Pro Glasses 2 (see below) cannot be worn with regular glasses. All eight reported not to suffer any inconvenience while walking across the festival grounds due to not wearing their usual corrective glasses.

Written informed consent was obtained from all participants prior to the start of the study. Participants were compensated for their participation with a coupon for one free drink at the festival. This research project does not belong to the regimen of the Dutch Act on Medical Research Involving Human Subjects, and therefore there is no need for approval of a Medical Ethics Committee. However, the study was approved by the Ethics Committee of the Faculty of Social and Behavioural Sciences of Utrecht University (protocol number 21-0348).

### Apparatus

Participants’ gaze was recorded using one of two Tobii Pro Glasses 2 (firmware versions 1.25.3-citronkola and 1.25.6-citronkola, respectively) and the Tobii Pro Glasses Controller (version 1.95.14258) running on two Windows 10 HP Pavilion X2 laptops. The Tobii Pro Glasses 2 recorded gaze at 50Hz and the scene camera recorded the view in front of the participant at 25Hz. The scene camera video includes an audio recording. The Tobii Pro Glasses 2 are standardly equipped with an accelerometer and gyroscope.

### Procedure

Participants were assigned to whoever of two experimenters was free at the moment. This experimenter fitted the participant with the Tobii Pro Glasses 2 and initiated the standard 1-point calibration procedure^2^. Hereafter, participants were asked to fixate 9 locations on a calibration-validation poster, so that we could estimate the accuracy for each recording. All participants that received instruction from one particular experimenter (author YdK, n = 33 participants) were instructed that they had to avoid eye contact as long as they were wearing the glasses and that they could not share this instruction with anyone. All other participants (n = 29 participants) received no such instruction from their respective experimenter (author JB).

**Fig. 1:**
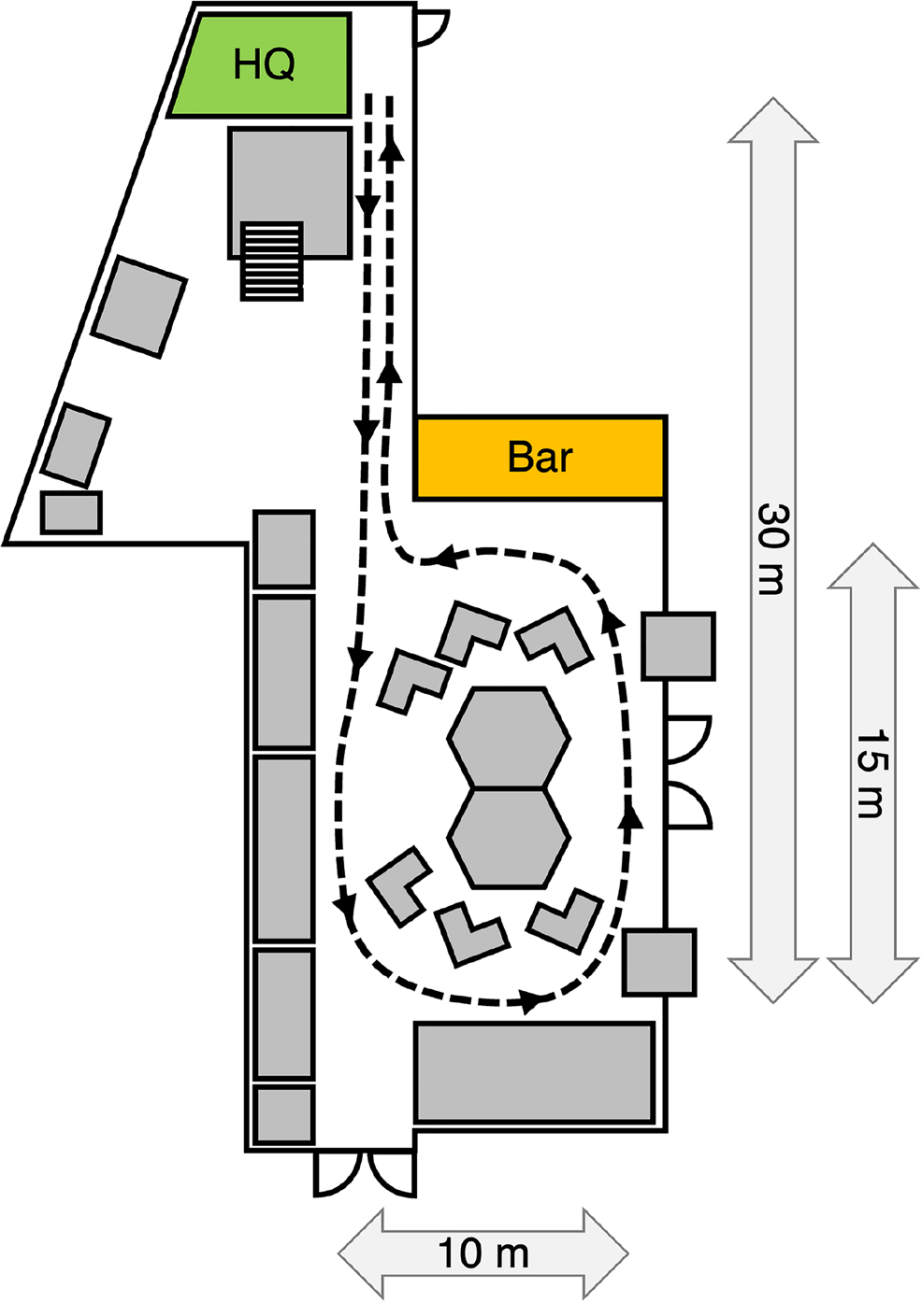
Schematic overview of the festival location and the route walked by the participants. The green area marked ‘HQ’ indicates the location at which participants received instructions and were fitted with the wearable eye tracker. The orange area indicates the bar that was used for a subset of the participants (see main body of article for details). Grey areas indicate other festival-related booths. The distances are approximations for the instructed route

Each participant was then transferred to a third experimenter who gave instructions on the route they had to walk (see Fig. 1). 9 out of the 62 participants were given a ticket for a free drink which they could get at the bar prior to returning the eye-tracking glasses. However, due to the fact that the lines at the bar were very unpredictable, this part of the instruction was skipped for the remaining participants. These participants received the ticket for a free drink upon returning to the experimenters.

After returning to the experimenters, the participants who received the instruction to avoid eye contact were asked how easy and/or comfortable they thought it was to follow this instruction. The eye-tracking glasses were then removed and the participant vacated the experimental booth.

### Data analysis

Participants who were instructed to avoid eye contact while walking their round may achieve this in a number of ways. First, they may orient their head differently in the world, for example by tilting their head towards the floor. Second, they may orient their eyes differently with respect to the eye-tracking glasses, for example by looking downward or by looking less at the head of other people they encounter. We outline below how we tackle each of these possibilities.

#### Video annotation

To describe gaze behavior during the route, we first had to annotate participants’ start and end times in the scene video. The start time was defined as the moment at which the participant walks away from the instructor explaining the route. The end time was defined as the moment at which the participant walks into the last part of the hallway (around the corner from the bar in Fig. 1) towards the instructor *or* the moment at which the participant joins a line for the bar *or* the moment at which a participant goes off route to do something else.

#### Pitch estimation

The Tobii Pro Glasses 2 is equipped with an inertial measurement unit (accelerometer and gyroscope). Using sensor fusion techniques (see Nazarahari & Rouhani, 2021, for an overview), pitch and roll orientations may be estimated from the accelerometer data (estimated angle of Earth’s gravitational force) and the gyroscope data (integration of the angular velocity measurements). Pitch orientation may be colloquially described as the tilt of the head forward towards, or upwards away from, the floor. Roll orientation may in this manner be described as the tilt of the head towards the left or right shoulder. For our purposes, the pitch orientation is thus most relevant, as it can indicate the degree to which participants look towards the floor to avoid eye contact.

Pitch estimation was achieved as follows. First, the accelerometer and gyroscope signals were resampled to 100 Hz using Gaussian smoothing (see e.g. van Leeuwen, Smeets, & Belopolsky, 2019), as they sample at different frequencies. For one eye tracker the gyroscope sampled at around 93 Hz and the accelerometer at around 103 Hz. For the other eye tracker, these sampling frequencies were around 95 Hz and 98 Hz, respectively. Second, a Kalman Filter implementation^3^ combined these signals to estimate pitch and roll orientation. For each participant, we then characterized the distribution of pitch angle throughout their round by its central tendency (median) and variation (inter-quartile range).

Note that Hyyti and Visala (2015) point out that pitch and roll estimation from inertial measurement units may be error-prone, resulting in both bias and gain errors. We also implemented their DCM method, which should be less error-prone. However, this method yielded large drifts in the beginning of recordings, and showed little difference with the Kalman Filter pitch estimation otherwise. We therefore used the Kalman Filter. What is most important for our purposes is that we compare median and inter-quartile ranges of the pitch angle distribution as well as a measure for overall angular velocity between groups, i.e. those that received the instruction to avoid eye contact and those who did not. Any bias or gain error should not matter much for the between-group comparison. We only assume that the pitch angle of the eye-tracking glasses with respect to the head, e.g. due to individual differences in head morphology, did not differ systematically between the groups.

#### Eye-tracking data analysis

Eye-tracking data were first used to determine distributions of gaze direction with respect to the eye-tracking glasses. This reveals whether participants who were instructed to avoid eye contact might have done so e.g. by looking down more with respect to the eye-tracking glasses. These distributions were determined for the azimuth and elevation components of a binocular signal (average of the left and right eye’s gaze signal) and then characterized by their central tendency (median) and variation (inter-quartile range). Secondly, eye-tracking data were used to determine where people looked in the world using manual mapping. This reveals whether participants who were instructed to avoid eye contact looked more or less at other people’s front, back, face or head. However, this analysis depends on adequate classification of slow-phases (or ‘fixations’, see Hessels, Niehorster, Nyström, Andersson, & Hooge, 2018b), which depends on the quality of the eye-tracking data obtained, specifically data loss and the variation in the gaze position signal. Thus, we first assessed the eye-tracking data quality and formulated exclusion criteria for slow-phase classification.

Eye-tracking data quality was operationalized using common measures of accuracy and data loss (see e.g. Holmqvist, Nyström, & Mulvey, 2012; Holmqvist et al.,, 2022), as well as the root mean square (RMS) sample-to-sample deviation of the gaze position signal (i.e. point of regard in the scene camera video) using a 300 ms moving window technique (see Hessels et al.,, 2020c, for details). The RMS deviation is often used as an operationalization of the precision of a recording for a non-moving observer fixating a static target in the world. Under these circumstances, large deviations are assumed to derive from the eye tracker (hardware and/or software, i.e. ‘variable error’), not the participant behavior. In our case, the values depend both on a variable error in the gaze-position signal and the eye movements made with respect to the eye-tracking glasses. However, the RMS deviation values are still indicative of the quality of the gaze-position signal and can be used to separate relatively good recordings from poor recordings. Moreover, high RMS deviations are particularly problematic for fixation classification, as outlined below. Data loss was operationalized as the percentage of samples without a valid gaze coordinate. Both the RMS deviation and data loss were extracted using the GlassesViewer software (Niehorster, Hessels, & Benjamins, 2020a), and computed for the period during which participants walked their round. Accuracy was operationalized as the angular distance between the location of the center of the 9 validation points and the location fixated by the participant. For each recording, author DN labeled the episode for which accuracy was estimated. Prior to computing the angular distance, the fixation location was mapped onto the plane of the validation poster using fiducial markers (see e.g. Niehorster et al.,, 2020b).

**Fig. 2:**
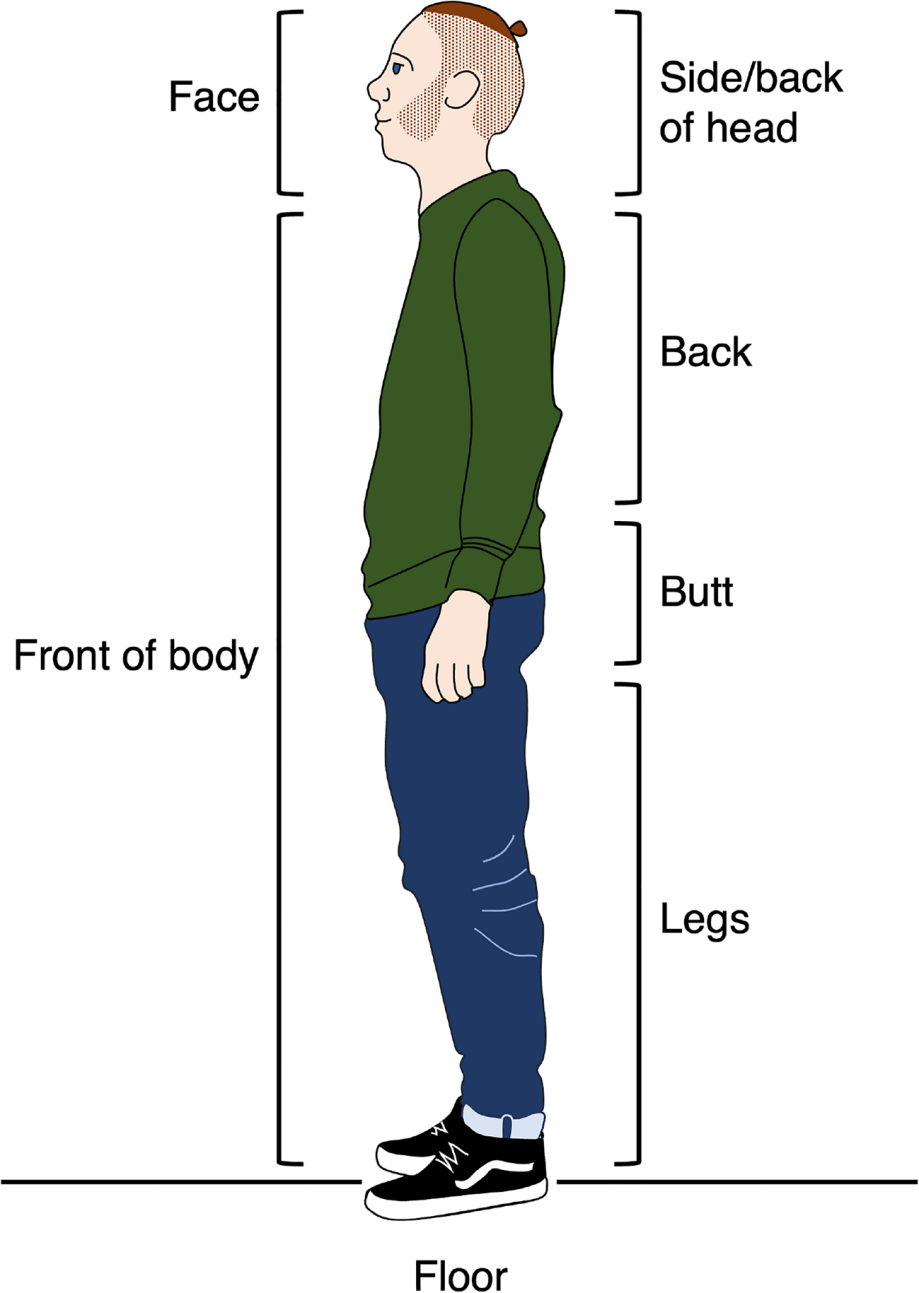
Overview of the categories to which fixations were mapped using GazeCode

Fixations were defined as “a period of time during which an area of the visual stimulus is looked at and thereby projected to a relatively constant location on the retina” (Hessels et al.,, 2018b, p. 21). Fixations were operationalized using a slow-phase classifier by Hessels et al., (2020c) with default settings implemented in GlassesViewer (Niehorster et al., 2020a). The fixation classifier is based on the adaptive-velocity threshold algorithm introduced by Hooge and Camps (2013). Each fixation that occurred within the walking round was then manually mapped in GazeCode (Benjamins, Hessels, & Hooge, 2018) to one of the following areas of interest (AOIs): frontal face, side or back of head, front of body, back, butt, legs, and floor (see Fig. 2). If a fixation was not on any of these AOIs, it was assigned the ‘non’-label. This categorization allows us to assess whether participants who were instructed to avoid eye contact looked less at the faces of others, and whether other gaze strategies were adopted. We decided upon a more fine-grained division of the back of other people’s bodies (head, back, butt, and legs) compared with the front (face, front of body) as we expected participants to more often find themselves following another person than encountering someone head on. This division allowed us to assess whether participants instructed to avoid eye contact generally look lower on the bodies of those people they follow.

#### Statistical analysis

We employ the same approach for statistical group-comparison as in our previous study (Hessels, Benjamins, van Doorn, Koenderink, & Hooge, 2021). That is, we report the Harrell-Davis estimated median and use nonparametric bootstrapping to compute the 95% confidence intervals around the median using the *decilespbci* MATLAB function provided by Rousselet, Pernet and Wilcox (2017). The number of bootstrap samples was set to the default value of 2000. These are supplemented by Bayesian statistical analyses conducted in JASP 0.16 (JASP Team, 2021) where appropriate. We use the notations for Bayes Factors as implemented in JASP and for interpretation of the values, we refer the reader to e.g. Table 1 in Schönbrodt and Wagenmakers (2018).

## Results

With our experiment, we aimed to determine whether people can avoid eye contact when navigating crowds and we investigated how this instruction affected gaze and/or walking behavior. One recording was inadvertently cut off before the participant started the round, yielding 61 usable recordings. Each recording consists of various data streams: accelerometer data, gyroscope data, gaze direction data and a video recording. For pitch estimation, the accelerometer and gyroscope data were used. For estimating the gaze direction distribution with respect to the eye-tracking glasses the gaze direction data were used. Finally, for manual mapping, gaze direction data and the video recordings were used. However, as we know that gaze estimation may suffer from make-up, poor lighting conditions, etc. (Holmqvist et al., 2022), we first determined the quality of the eye-tracking data and formalized exclusion criteria for the eye-tracking data analyses.

### Eye-tracking data quality and exclusion

As noted, eye-tracking data quality was operationalized using measures for data loss and accuracy, and the RMS deviation of the gaze position signal. For our purposes, the RMS deviation of the gaze position signal and data loss are the limiting factors in fixation classification, while high accuracy is most relevant for correctly mapping fixations to locations in the world. We thus first consider exclusion based on data loss and RMS deviation of the gaze position signal for fixation classification.

**Fig. 3:**
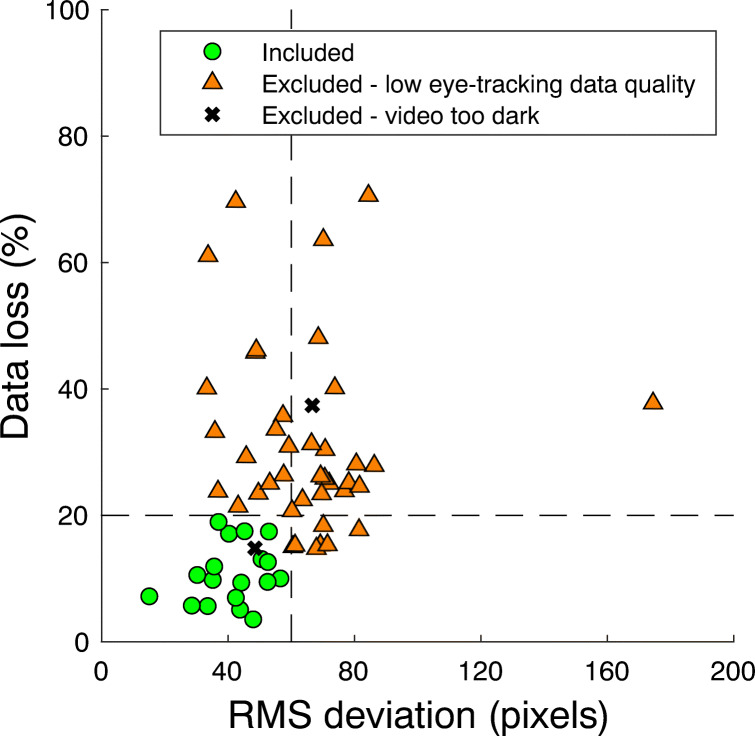
Eye-tracking data quality and exclusion criteria for slow-phase (fixation) classification. Data quality was operationalized as the percentage data loss and the root mean square (RMS) sample-to-sample deviation of the binocular gaze position signal. Recordings were excluded (orange triangles) from slow-phase classification if the RMS deviation exceeded 60 pixels or data loss exceeded 20%. Two additional recordings, indicated by the black crosses, were excluded because the video was so dark it could not be used for manual mapping of slow phases (see main body of text)

Figure 3 depicts data loss and RMS deviation for each participant. Comparing this to our previous work on looking behavior during locomotion with the same eye tracker, we observe much lower data quality (high RMS deviation and percentage data loss) in the present study (compare Figure 6 in Hessels et al.,, 2020a). Note that we attribute these differences in RMS deviation between the studies to data quality, not participant behavior (i.e. more eye movements with respect to the head), as the participants were asked to do the same thing (walk around). To determine which recordings were suitable for fixation classification, the fixation classification results were checked for each recording in GlassesViewer (Niehorster et al., 2020a) by authors RH and SvH. Their subjective assessment of which recordings were not suitable for fixation classification, even after trying to tweak the fixation classifier for each recording, matched well with the exclusion criteria originally introduced by Hessels et al., (2020a), namely excluding recordings with more than 20% data loss or more than 60 pixels RMS deviation. This led to the exclusion of 42 participants from fixation classification. Furthermore, it turned out that for one additional recording, the scene camera video was so dark, almost nothing was visible for manual mapping of fixations to locations in the world.^4^ A final set of 18 recordings was used for fixation classification (8 who received the instruction to avoid eye contact, 10 who received no additional instruction). Note that an equal number of females and males were left for this analysis (9 per gender).

For one recording, the calibration-validation procedure was missing from the video, as the recording was restarted after the validation due to technical complications. For the other 60 recordings, median accuracy was 0.69^∘^, with a range of 0.08–7.31^∘^. The large range was due to large inaccuracies for just three recordings, which were already excluded for fixation classification based on the RMS deviation and/or data loss criteria. We therefore did not exclude any additional recordings. 68% of all recordings had an accuracy below 1^∘^, 90% of the recordings below 2^∘^, and 95% of the recordings below 3^∘^. Median accuracy for the 18 recordings included for fixation classification was 0.79^∘^ (range = 0.29–2.17^∘^).

The full set of 61 recordings was used for the distributions of pitch orientation and gaze direction with respect to the eye-tracking glasses described below. The reduced set of 18 recordings was used for manual mapping of gaze to the world. However, in the below analyses we highlight which recordings were of sufficiently high eye-tracking data quality according to the criteria for RMS deviation of the gaze position signal and data loss described above, so that the reader may judge whether the other measures (pitch orientation and gaze direction with respect to the eye-tracking glasses) seem to relate to eye-tracking data quality.

### Walking times

For the 61 participants for whom the recording was successful, round completion (i.e. walking time) took between 47 and 260 s. There did not seem to be any difference in round completion times between the group instructed to avoid eye contact (n = 33, median = 91.91 s, 95%CI = 79.90–109.92 s) and the group who received no further instructions (n = 28, median = 91.29 s, 95%CI = 75.73–111.60 s). Overall, participants followed the instructed route well, although some of the participants deviated briefly off route by walking through the doors on the right-hand side of Fig. 1. This occurred for two participants from each group.

### Head orientation as a function of instruction

**Fig. 4:**
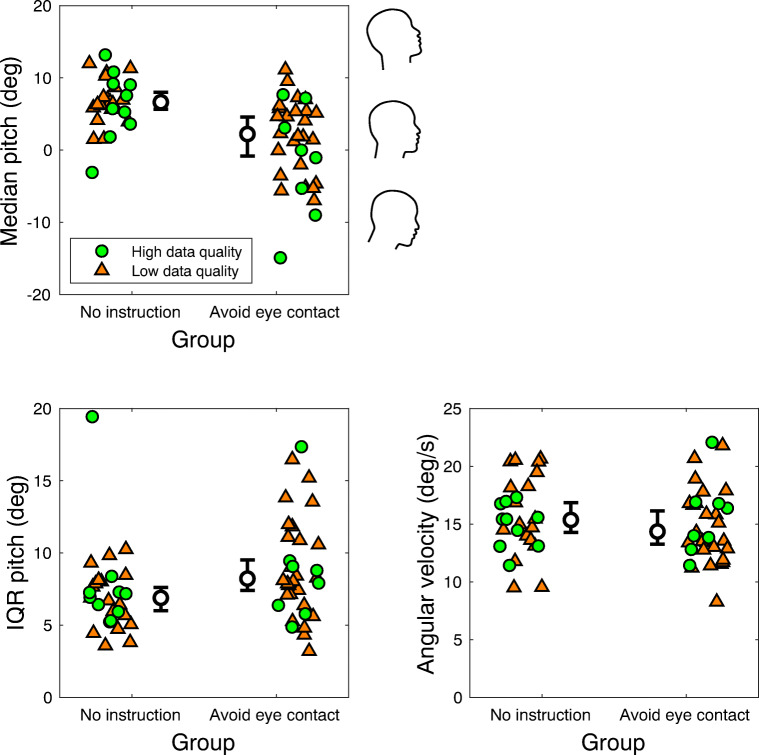
Median pitch orientation, variation in pitch orientation, and angular velocity along the pitch axis as a function of instruction (avoid eye contact or no specific instruction) when navigating crowds. Note that the pitch orientation represents orientation of the eye tracker in the world. Each colored marker represents the median (top left panel), inter-quartile range (IQR; bottom left panel), or angular velocity (bottom right panel) for one participant. The zero for the pitch orientation is when the eye-tracking glasses are tilted 4 degrees forward with respect to the world, as a result of how the gyroscope and accelerometer are mounted in the eye tracker. Lower values indicate forward head pitch, i.e. towards the floor. Green circular markers represent those participants with eye-tracking data quality sufficient for fixation classification, orange triangle markers those with insufficient data quality. No relation between eye-tracking data quality and measures of pitch orientation is to be expected, as pitch orientation is derived from accelerometer and gyroscope data only. Indeed there does not seem to be a systematic difference between the high and low eye-tracking data quality recordings. The inner black circle represents the Harrell-Davis estimated median of the group, with error bars depicting the bootstrapped 95% confidence interval of the median. Note that the colored markers are jittered in horizontal direction for visualization purposes only

Do ‘eye-contact avoiders’ show similar distributions of pitch orientation as the participants without additional instructions? We characterized the head orientation along the pitch axis for the two groups by (1) determining the central tendency (median) of the eye tracker pitch orientation in the world, (2) the variance (IQR), and (3) a measure of distance travelled divided by the walking time (i.e. overall angular velocity along the pitch axis). The latter is computed as the sum of inter-sample orientation differences divided by the walking time. The central tendency reveals whether participants who were instructed to avoid eye contact pitched their head forward compared with the ‘no instruction’ group. The IQR reveals whether the two groups differed in the range of orientations along the pitch axis, and the angular velocity reveals whether the two groups differed in the amount of head movements made along the pitch axis. Figure 4 depicts the median pitch orientation, variation in pitch orientation, and angular velocity along the pitch axis as a function of instruction.

The median pitch orientation for the ‘avoid eye contact’ group seems substantially lower (median = 2.22 deg, 95%CI -0.84–4.58 deg) than for the ‘no instruction’ group (median = 6.63 deg, 95%CI 5.67–8.02 deg). We corroborated this with a Bayesian independent samples t-test, with a BF_10_ of 257.54 for the hypothesis that the median pitch orientation was not equal for the ‘avoid eye contact’ and ‘no instruction’ group. While the IQR of the pitch orientation seemed slightly higher for the ‘avoid eye contact’ group (median IQR = 8.23 deg, 95%CI 7.40–9.51 deg) than for the ‘no instruction’ group (median IQR = 6.89 deg, 95%CI 6.01–7.62 deg), a Bayesian independent-samples t-test yielded a BF_10_ of 1.39 for the hypothesis that the IQR was not equal between the groups. This indicates no clear evidence in favor or against this hypothesis. The angular velocity along the pitch axis was roughly equal for both groups: For the ‘avoid eye contact’ group the median angular velocity along the pitch axis was 14.36 deg/s (95%CI 13.25–16.15 deg/s), for the ‘no instruction’ group the median angular velocity along the pitch axis was 15.36 deg/s (95%CI 14.27–16.85 deg/s). Thus, participants who were instructed to avoid eye contact pitched their head substantially more towards the floor, but did not seem to change their head pitch more (i.e. by making more or substantially larger head movements in the upward/downward direction) than participants who did not receive any additional instructions.

### Gaze direction as a function of instruction

**Fig. 5:**
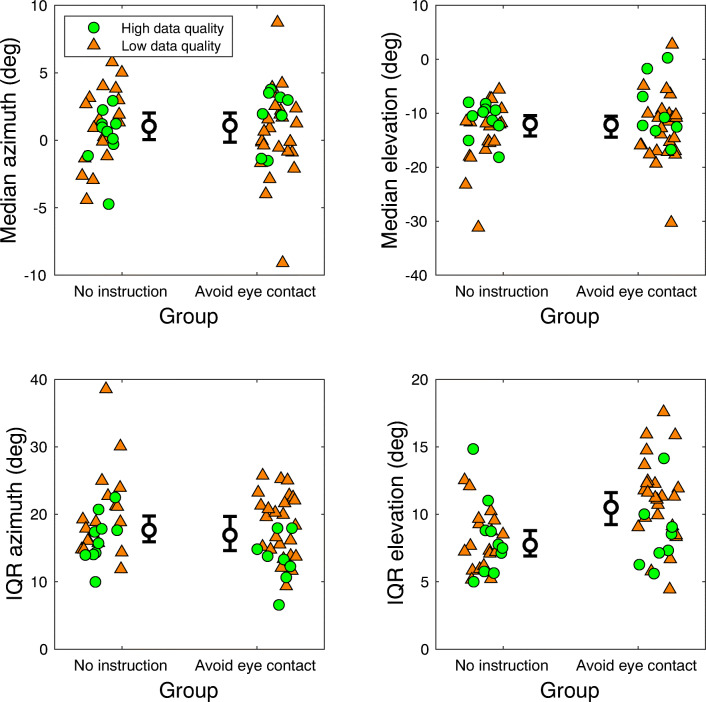
Median and variation in gaze direction relative to the head as a function of instruction (avoid eye contact or no specific instruction) when navigating crowds. Gaze direction is separated in azimuth (left panels) and elevation (right panels) components of the binocular signal in Fick coordinates (Haslwanter, 1995) with respect to the center of the scene camera of the Tobii Pro Glasses 2. Each colored marker represents the median (top panels) or inter-quartile range (IQR; bottom panels) for one participant. Green circular markers represent those participants with eye-tracking data quality sufficient for fixation classification, orange triangle markers those with insufficient data quality. The inner black circle represents the Harrell-Davis estimated median of the group, with error bars depicting the bootstrapped 95% confidence interval of the median. Note that the colored markers are jittered in horizontal direction for visualization purposes only

Do ‘eye-contact avoiders’ show similar distributions of gaze direction with respect to the eye-tracking glasses as the participants without additional instructions? Figure 5 depicts the median and variation in gaze direction (azimuth and elevation components) as a function of instruction. The median and boostrapped 95% confidence intervals (black circles with error bars) for the ‘avoid eye contact’ and ‘no instruction’ groups overlap almost completely for the median azimuth (top left panel), median elevation (top right panel) and inter-quartile range (IQR) of the azimuth (bottom left panel). However, the median IQR for the elevation component was higher for the ‘avoid eye contact’ group (median IQR = 10.52 deg, 95%CI 9.24–11.60 deg) than for the ‘no instruction’ group (median IQR = 7.72 deg, 95%CI 6.91–8.80 deg). We corroborated this with a Bayesian independent samples t-test, with a BF_10_ of 15.29 for the hypothesis that the IQR of the elevation component was not equal for the ‘avoid eye contact’ and ‘no instruction’ group. This shows that for the group instructed to avoid eye contact the distribution of gaze direction in the elevation component was substantially broader. For a person with an upright head (i.e. not oriented towards the left or right shoulder), this means more variance in looking in the upward-downward direction. One may argue that the difference in the IQR for the elevation component would not hold for the green markers alone (i.e. those recording with high eye-tracking data quality). However, if low data quality predicts a larger IQR for the elevation component, one would expect the same pattern of higher IQRs for the orange markers in the group that received no additional instruction. Thus, the present analysis combined with the differences in the pitch orientation described above, suggests that participants instructed to avoid eye contact looked towards the floor more and changed their gaze direction upward/downward more with respect to the eye-tracking glasses.

### Gaze location in the world as a function of instruction

For the 18 participants with high quality eye-tracking data, fixations were mapped using GazeCode (Benjamins et al., 2018) to one of the following areas of interest (AOIs): face, front of body, side or back of head, back, butt, legs, or floor (see Fig. 2). A ‘non’-label was assigned to all fixations that could not be mapped to any of the other AOIs. The mapping procedure was completed by a coder naive to the purposes of the experiment. To determine the inter-rater agreement, one of the authors (SvH) also completed the manual mapping of fixations for all recordings. Another author (NV) prepared the recordings such that author SvH did not see the instruction procedure and did not see which group the participant belonged to.

We estimated the inter-rater agreement using Cohen’s kappa, which was 0.58 across all recordings. Confusion matrices revealed that the naive coder was more likely to not code a fixation (thus receiving the ‘non’-label). Without the ‘non’ category included, Cohen’s kappa was 0.68. According to Landis and Koch (1977), this indicates moderate to substantial agreement. Moreover, the inter-rater agreement we observed is in the same range as that observed in previous studies on looking behavior to other people during locomotion (Fotios, Uttley, & Fox, 2018; Hessels et al.,, 2020c). Importantly, the conclusions we draw below do not depend on who’s codings we take, either from the naive or informed coder.

**Fig. 6:**
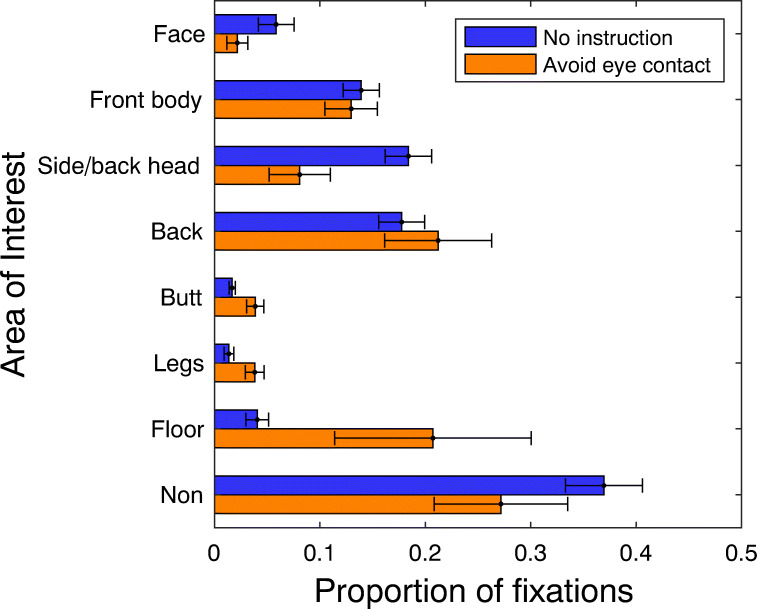
Gaze location in the world as a function of instruction (no additional instructions or avoid eye contact). Proportion of fixations on each area of interest: face, front of body, side or back of head, back, butt, legs, floor or non (other location or could not be determined). Error bars indicate standard error of the mean. In contrast to our previous analyses, we do not report medians and bootstrapped 95% confidence intervals here, as there are too few data points (8 or 10 per group) for the bootstrapping procedure (see Rousselet et al.,, 2017, p. 1747)

Figure 6 depicts the proportion of fixations that occurred for each AOI. Approximately 50–60% of all fixations were directed at other people (face, front body, side/back head, back, butt, or legs). This corresponds well with the average proportion of gaze on passersby during single encounters (cf. Hessels et al.,, 2020a, figure 8). Although the overall proportion of fixations on the face appears low (less than 10%), we observed clear differences in the distribution of gaze between the two groups: Participants who were instructed to avoid eye contact looked less at the faces or heads and more at the butt and legs of other people, and looked more at the floor than those who did not receive additional instructions. We corroborated this statistically using a Bayesian repeated-measures ANOVA on the proportion of fixations to the various AOIs, with AOI (face, front of body, side or back of head, back, butt, legs, floor or non) as a within-subjects factor and group (no instruction, avoid eye contact) as a between-subjects factor. The model including both the AOI and group term, as well as the interaction term (AOI * group), was best supported by the data (BF_m_ = 14.68). Note that the pattern is highly similar if we consider the relative total looking time instead of the proportion of fixations. Thus, participants instructed to avoid eye contact gazed at different areas of the world. This is consistent with our observation that the participants who were instructed to avoid eye contact pitched their head more towards the floor than the other participants. As a result, fixation locations are further down in the world: less at heads, more at the lower body and floor.

### Practical considerations

The results described above show convincingly how people instructed to avoid eye contact achieve this while navigating crowds. Importantly, this was despite many unforeseen circumstances encountered during the experiment. We outline the most notable circumstances below, as they may help researchers conceive and setup their future wearable eye-tracking experiments with real crowds or in public environments.

#### No control over lighting outside calibration area

Ambient lighting may affect eye-tracking data quality (see e.g. Blignaut & Wium, 2014). As such, we brought studio lighting to the festival hall for use during setup, calibration, and validation. However, we had no control over the lighting in the rest of the event hall, through which participants walked their round. When participants were immersed in the crowds, it was substantially darker than during calibration and validation. We quantified the effect of this on pupil size, by computing the 2nd and 98th percentiles as estimates of minimum and maximum pupil diameter. The average minimum pupil size was 3.06 mm across all participants, the average maximum pupil diameter was 5.89 mm, yielding a mean difference of 2.83 mm. Crucially, changes in pupil size may affect the accuracy of the gaze direction signal as a result of the pupil-size artifact (e.g. Drewes, Zhu, Hu, & Hu, 2014; Wyatt, 2010). Hooge, Hessels, and Nyström, (2019a), for example, observed an apparent change in the vergence angle of − 0.36^∘^ per mm pupil diameter change for the SR Research EyeLink 1000+ and − 0.72^∘^ per mm pupil diameter change for the SMI iView X Hi-Speed 1250. Thus, one might expect that substantial non-eye movement related changes in the vergence angle may have occurred in our study. However, it is unknown whether the pupil-size artifact occurs for the Tobii Pro Glasses 2 and, if so, what the magnitude and inter-individual variability are. In addition, previous research has shown that this artifact also has a viewing-direction dependent component (Hooge, Niehorster, Hessels, Cleveland, & Nyström, 2021), which may add to the inaccuracy of the gaze direction signal as a result of pupil-size changes. What is most crucial, is that we do not know how any pupil-size related inaccuracy may affect the projection of the gaze point on the scene camera image, which we use here for mapping gaze to the world. Clearly, research using wearable eye trackers in situations where lighting conditions may vary substantially would benefit from future research on this topic.

#### Little to no control over crowd sizes and route

Due to the nature of the festival—an event with multiple scheduled lectures, performances, and ongoing experiments—it was impossible to control or even predict the size of the crowds that our participants encountered. Crowd sizes may have varied over the course of the evening, and over the course of each round. We observed a similar problem while conducting pilot experiments for one of our previous studies (Hessels et al., 2020c), for which we ended up staging our own crowds over which we had full control. This may have affected e.g. the proportion of fixations directed at other people across our participants.

Prior to the festival we had no idea which route we were going to ask our participants to walk. Even though the layout of the event hall was already decided upon, last minute changes and any placed decor may affect how we were going to instruct the participants. Moreover, some participants missed a turn, and inadvertently walked a longer route than others, which may partly explain the large range in walking times described above.

#### Little control over participant behavior

Participants do not always behave as one instructs or expects. For example, we had two wearable eye trackers, which allowed us to conduct two recordings simultaneously. Although we planned to send out each participant once they received their instructions, it turned out quickly that friends and family started waiting for each other so they could walk together. Thus, some participants walked on their own, while others walked in pairs. Another example is that not everyone instructed to avoid eye contact kept this instruction a secret—although they were explicitly told to do so. Some participants mentioned this to their friend immediately upon leaving the experimenters, others revealed it later or only indirectly (e.g. by asking ‘did you get an assignment too?’). Finally, we noticed that some participants fell out of their role as an experimental subject upon encountering friends or acquaintances. One particularly interesting case was a participant instructed to avoid eye contact who came across a friend. She looked the friend directly in the face, explained what she was doing and revealed which instruction she was given. Once she went back to walking her round, she switched back into her role of experimental subject, avoiding eye contact and keeping the instruction secret from the other friend walking beside her. This indicates that for experiments with little constraint on participant behavior, it is essential to assess whether participants followed the instructions well.

#### Evidence on people being aware of the eye tracker or not

Previous research suggests that people gaze differently, e.g. show socially desirable behavior, when they know their gaze is being recorded (e.g. Risko & Kingstone, 2011). Indeed, the audio recordings revealed that some participants felt they were being watched and/or were very aware of where they were looking. Conversely, some participants exhibited behavior that suggests the opposite. For example, they unlocked their phones in plain view, and started sending text messages to other people. Either they were unaware at the time that the researchers would later see what they were doing, or they simply did not care about it. It may be interesting for future research to investigate these behaviors and how they may relate to where people look in the world. Do these participants show a less socially desirable gaze pattern, for example?

## Discussion

We determined empirically whether humans can avoid eye contact while navigating through crowds and investigated how this affected gaze and/or walking behavior. To this end, we conducted a wearable eye tracking experiment with 62 participants walking a route at a popular science festival, approximately half of which were instructed to avoid eye contact. In addition, we shared the practical problems we encountered when conducting this study to aid researchers conceiving and setting up future experiments in this relatively young research field.

Regarding eye contact avoidance, we found that participants instructed to avoid eye contact pitched their heads more towards the floor and changed their gaze direction upward/downward more with respect to the eye-tracking glasses than participants who received no additional instructions. This resulted in the participants instructed to avoid eye contact looking less at the face and head and more towards lower areas in the world (butt, legs, and floor). Importantly, we did not observe any differences in walking times, which suggests that walking behavior was not severely hampered by the ‘avoid eye contact’ instruction. However, it should be noted that the range in walking times was substantial, with some participants walking five times as long as others. Thus, eye contact can be avoided in crowds, i.e. it is possible to override a potential bias to make eye contact. It is further interesting to note that many, but not all, participants mentioned after the experiment that they felt it quite difficult or uncomfortable to avoid eye contact. Combined with our previous research (Hessels et al., 2020c), this suggests that humans can flexibly allocate their gaze when navigating crowds. They can effectively avoid making eye contact, or look more at others to seek out social affordances in crowds (Hessels et al., 2020c), without hampering crowd navigation. One expects that such flexibility may be less for crowds in which there is substantial pressure on navigation time and where there are many people to be avoided, for example during evacuations. In this regard, the absence of such flexibility in gaze behavior may be a relevant measure to consider in evacuation dynamics (e.g. Kitazawa & Fujiyama, 2010), as it could indicate that every fixation is necessary to safely navigate.

Besides what our results reveal about eye contact and crowd navigation, what might be learned about generic and/or specific patterns of gaze during fleeting encounters? One interesting finding, is that our participants solved the ‘avoid eye contact’ instruction mainly with their heads and eyes, not their eyes alone. That is, they oriented their head more towards the floor, but did show a different distribution of gaze direction with respect to the eye-tracking glasses, compared with the participants who received no additional instruction. This pattern has been observed in other wearable eye-tracking studies as well. Foulsham et al., (2011), for example, reported that for people walking across campus, most locations on the horizon were oriented towards not with the eyes only (i.e. by making an eye movement, but keeping one’s head oriented in the same direction), but with both the head and eyes. More generally speaking, it has been observed that orientation to targets in the world is solved mostly by orientations of the body and head, not the eyes (Radau, Tweed, & Vilis, 1994). Why might one expect this to be different for avoiding eye contact? For one, making eye contact may have consequences for subsequent social interaction: Where one looks can be a signal for someone else to e.g. initiate a conversation, or to avoid one altogether (see e.g. Hessels et al.,, 2020a; Laidlaw et al.,, 2011). Given that head orientation is more easily estimated than gaze direction from peripheral vision (Loomis, Kelly, Pusch, Bailenson, & Beall, 2008, see also Hessels, 2020, p. 8600–861), orienting one’s eyes instead of one’s head away may help obscure active eye-contact avoidance.

How the head and eyes are oriented in the world in social situations is also interesting from a comparative perspective. Kobayashi and Kohshima (1997), for example, examined the width to height ratio of eyes, the color of the sclera and the exposed sclera size of primates. They reported that what distinguishes humans from non-human primates is the white sclera, the amount of visible sclera and a large width to height ratio of the eye opening, which results in eyes whose orientation can easily be judged by others. They suggest that a “gaze-signal enhancement might aid the communication required for increased cooperative and mutualistic behaviours to allow group hunting and scavenging. A small change in sclera coloration may have altered ‘gaze-camouflaged’ to ‘gaze-signalling’ eyes” (p. 768). A dark sclera, on the other hand, may help obscure gaze direction and be considered adaptive, for example in foraging situations where one’s gaze direction may be exploited by a conspecific (Hall et al., 2014). Yet, there is still debate around the functional use of dark sclera. Mayhew and Gómez (2015), for example, found that many Gorillas also have white exposed sclera, which challenges the assumed widespread ‘gaze camouflaging’ function of dark sclera for non-human primates. With regard to comparative differences in the use of another person’s head or eye orientation, Tomasello, Hare, Lehmann and Call (2007) concluded that great apes followed a human’s gaze direction mainly on the basis of head orientation, while human infants mainly followed the human’s gaze direction based on eye orientation. Our results suggest that instead of focusing only on comparative differences between humans and non-human primates in the morphology of eyes or the use of head/eye orientation, it is also relevant to understand when the eye orientation may deviate from the head orientation and in what social situations. When are the eyes oriented to a different location in the world than the head? And what are the opportunities for subsequent social interaction? One could conceive of studies similar to e.g. Jayaraman, Fausey and Smith (2015), to reveal (developmental) patterns in the availability of distinct head and eye orientations in one’s environment and the corresponding social affordances.

Regarding the practical considerations, we revealed a number of potential problems for wearable eye-tracking studies in public environments. First, we reported that data quality was much worse (higher RMS deviation of the gaze position signal, more data loss) than in our previous studies using the same eye trackers and a comparable research question. This was particularly problematic for fixation classification and mapping of fixations to the world, i.e. at what locations in the world did participants look? The exclusion rate was roughly 69% in the present study, while in our previous work it was only 13% (Hessels et al., 2020a). We highlighted one potential source of this lower data quality, namely the large pupil size or large pupil size changes after calibration (Holmqvist et al., 2022). We welcome studies on the relation between pupil size and data quality for wearable eye trackers, particularly regarding the effects of the pupil-size artifact (e.g. Hooge et al.,, 2019a). Moreover, it seems that the development of noise-robust fixation classification algorithms for wearable eye-tracking studies may improve the inclusion rates. Such algorithms were also developed for eye-tracking data from infant participants recorded with world-bound (i.e. remote) eye trackers (Hessels, Niehorster, Kemner, & Hooge, 2017; Renswoude et al.,, 2018). The eye-movement measures derived from these algorithms are less susceptible to the variability in data quality observed with infant participants, and allow e.g. more reliable between-group and between-age comparisons, which are crucial to developmental research. Finally, in our study, the inclusion rate for fixation classification was much lower for females (9/40) than for males (9/22). One hypothesis is that proportionally more make-up use among females causes this difference. Dark make-up may interfere with gaze estimation, particularly when dark eye lashes partially cover the (large) pupil (see e.g. figure 4.5 in Holmqvist et al.,, 2011).

To what degree do these data quality issues affect our conclusions, or the design of future studies? The eye-tracking data quality mainly limited what information we could obtain from the individual recordings, where fixation classification was not possible for those recordings with low data quality. Future studies with wearable eye trackers that are conducted in similar circumstances thus need potentially large group sizes if the exclusion rates are so high. An important open question is whether data quality is also related systematically to some of the behaviors we investigate. We have assumed not, but it may be the case. For example, pupil size changes after calibration might be larger for those people that are more aroused because of some instruction to make or avoid eye contact. Yet, we also observed differences in head orientation along the pitch axis and the distribution of gaze direction with respect to the eye-tracking glasses. Such measures may be termed global measures, in the sense that they can be computed without regard for the specific object or person at which one looks in the world (see e.g. Dowiasch, Marx, Einhäuser, & Bremmer, 2015, for a similar example). Such global eye-tracking measures are potentially less affected by data quality, e.g. because they do not rely on fixation classification. Thus, if global measures can be used to answer a research question, they might make certain studies more feasible.

A second problem we reported was that we had little control over the environment, specifically the sizes of the crowds that participants walked through and the exact route that they followed. Moreover, some participants were quick to reveal the instruction they had been told not to reveal, while others did keep it a secret. How might these issues have affected our results? For one, the substantial variability in walking times may mean that any difficulties that participants encountered in navigating crowds due to the ‘avoid eye contact’ instruction may not have been picked up. One would likely need an experiment tailored specifically to how and when avoiding other people (at every encounter) may be hampered when one cannot make eye contact. The fact that people differed in whether they revealed (or followed) the instructions faithfully, may have affected the variability in behavior that we observed. It would be interesting to determine whether the gaze or walking behavior was related to how easy participants found it to follow the ‘avoid eye contact’ instruction. However, we did not assess this systematically. We advise future studies to take our considerations into account when designing and validating the instructions or tasks given to participants in relatively unconstrained environments.

Our study was set up to investigate whether humans can avoid eye contact while navigating through crowds. For this, we used crowds as they naturally occurred at a popular science festival. While such unconstrained crowds are useful in that they may be representative for the kinds of crowds humans may encounter in their daily life, they pose a number of challenges for more fine-grained analyses. For example, at any moment in time there may be multiple people at various distances from the participant, and from moment to moment multiple people may appear in and disappear from view. Thus, if one is interested in the relation between gaze behavior, the (dis)appearance of people in/from view and interpersonal distances, one might have to manually annotate hours of video. Such manual annotation might further require high-quality videos (not too dark and little motion blur) to estimate all the necessary variables. Some variables may not even be annotatable from the scene camera videos. For example, a person that appears in view of the scene camera might have been visible to the participant already. For these kinds of analyses, one might instead wish to set up dedicated studies where e.g. crowd size, crowd behavior, appearance into view, and/or interpersonal distances are under one’s control (e.g. Hessels et al.,, 2020a, 2020c). We advise researchers interested in this topic to carefully consider which variables they might want to control, and which they might wish to leave unconstrained.

To conclude, humans can flexibly allocate their gaze to the environment while navigating crowds and avoid eye contact by orienting their head and eyes towards the floor, not by orienting only their eyes downward with respect to the head. This behavior may be relevant in a comparative perspective. Furthermore, practical considerations with regard to data quality, control of the environment, and participant adherence to instruction are important for wearable eye-tracking studies in unconstrained environments. Researchers should carefully consider these when conceiving similar field studies. In particular, global eye-tracking measures and head orientation may be fruitful when eye-tracking data quality is low.

## Footnotes

1 Depending on the exact operationalization of eye contact, this requires one or two eye trackers. When eye contact is operationalized as looking at the face or eye area of another person, one eye tracker suffices. If eye contact is operationalized as mutual looking at the face or eye region, two eye trackers are required. See Jongerius et al., (2020) and Valtakari et al., (2021) for more in-depth discussions.

2 See https://www.tobiipro.com/siteassets/tobii-pro/user-manuals/tobii-pro-glasses-2-user-manual.pdf for details

3 By https://github.com/pms67/Attitude-Estimation

4 This also applied to 2 other recordings, one of which was the recording that was cut short before the participant commenced the round and another which was already excluded based on eye-tracking data quality exclusion criteria.
